# Whole-genome sequencing for One Health surveillance of antimicrobial resistance in conflict zones: a case study of *Salmonella* spp. and *Campylobacter* spp. in the West Bank, Palestine

**DOI:** 10.1128/aem.00658-23

**Published:** 2023-09-28

**Authors:** Said Abukhattab, Salome Hosch, Niveen M. E. Abu-Rmeileh, Shadi Hasan, Pascale Vonaesch, Lisa Crump, Jan Hattendorf, Claudia Daubenberger, Jakob Zinsstag, Tobias Schindler

**Affiliations:** 1 Swiss Tropical and Public Health Institute, Allschwil, Switzerland; 2 University of Basel, Basel, Switzerland; 3 Institute of Community and Public Health, Birzeit University, Birzeit, Palestine; 4 Master program in Clinical Laboratory Sciences, Birzeit University, Birzeit, Palestine; 5 Department of Fundamental Microbiology, University of Lausanne, Lausanne, Switzerland; Centers for Disease Control and Prevention, Atlanta, Georgia, USA

**Keywords:** Palestine, *Campylobacter*, *Salmonella*, AMR, WGS, One Health

## Abstract

Antimicrobial resistance (AMR) is a critical global concern driven by the overuse, misuse, and/or usage of inadequate antibiotics on humans, animals’ agriculture, and as a result of contaminated environments. This study is the first One Health survey in the Middle East that incorporated whole-genome sequencing (WGS) to examine the spread of AMR in *Campylobacter* spp. and *Salmonella* spp. This cross-sectional study was conducted to examine the role of AMR at the human-animal-environmental interface and was performed in Ramallah/Al-Bireh and Jerusalem governorates of the central West Bank, Palestine. In 2021 and 2022, a total of 592 samples were collected and analyzed. From a total of 65 *Campylobacter jejuni* and 19 *Salmonella* spp. isolates, DNA was extracted for WGS using Oxford Nanopore Technologies MinION platform. We found that the dominant serotypes of *C. jejuni* and *Salmonella enterica* were present in chicken manure, chicken meat sold in markets, and feces of asymptomatic farm workers, with high genetic similarities between the isolates regardless of origin. Additionally, our results showed rapid strain turnover in *C. jejuni* from the same sites between 2021 and 2022. Most of the positive *Salmonella* spp. samples were multidrug-resistant (MDR) *S. enterica* serovar Muenchen carrying the plasmid of emerging *S. infantis* (pESI) megaplasmid, conferring resistance to multiple antibiotics. Our findings highlight the spread of MDR foodborne pathogens from animals to humans through the food chain, emphasizing the importance of a One Health approach that considers the interconnections between human, animal, and environmental health.

**IMPORTANCE:**

Prior to this study, there existed hardly an integrated human-animal-environmental study of Salmonellosis and Campylobacteriosis and related AMR in Middle Eastern countries. The few existing studies lack robust epidemiological study designs, adequate for a One Health approach, and did not use WGS to determine the circulating serotypes and their AMR profiles. Civil unrest and war in Middle Eastern countries drive AMR because of the breakdown of public health and food security services. This study samples simultaneously humans, animals, and the environment to comprehensively investigate foodborne pathogens in the broiler chicken production chain in Palestine using WGS. We show that identical serotypes of *C. jejuni* and *S. enterica* can be found in samples from chicken farms, chicken meat sold in markets, and asymptomatic broiler chicken production workers. The most striking feature is the rapid dynamic of change in the genetic profile of the detected species in the same sampling locations. The majority of positive *Salmonella* spp. samples are MDR *S. enterica* serovar Muenchen isolates carrying the pESI megaplasmid. The results demonstrate a close relationship between the *S. enterica* serovar Muenchen isolates found in our sample collection and those responsible for 40% of all clinical *Salmonella* spp. isolates in Israel as previously reported, with a sequence identity of over 99.9%. These findings suggest the transboundary spread of MDR *S. enterica* serovar Muenchen strains from animals to humans through the food chain. The study underscores the importance of combining integrated One Health studies with WGS for detecting environmental-animal-human transmission of foodborne pathogens that could not be detected otherwise. This study showcases the benefits of integrated environmental-animal-human sampling and WGS for monitoring AMR. Environmental samples, which may be more accessible in conflict-torn places where monitoring systems are limited and regulations are weak, can provide an effective AMR surveillance solution. WGS of bacterial isolates provides causal inference of the distribution and spread of bacterial serotypes and AMR in complex social-ecological systems. Consequently, our results point toward the expected benefits of operationalizing a One Health approach through closer cooperation of public and animal health and food safety authorities.

## INTRODUCTION

The global spread of antimicrobial resistance (AMR), often referred to as the “silent pandemic” is one of the major challenges to public health in the 21st century. The O’Neill report estimates that by 2050, 10 million lives could be lost annually due to AMR (1). The problem of AMR is exacerbated in conflict-torn regions, such as the Middle East, where overuse of antibiotics, fragmented monitoring systems, inadequate infrastructure, and a lack of regulations and controls contribute to the rise of AMR. This includes both community and nosocomial transmission, which raises the incidence of AMR in these areas (2). Evidence is accumulating that prolonged and intense conflicts, as in the Palestine territories, lead to social and environmental conditions that foster the emergence of AMR (3). In Palestine, the lack of comprehensive national surveillance for AMR and weak published data hinder the ability to assess the extent of the problem and its risk factors. Furthermore, as a religious tourist destination, Palestine poses a potential risk for the emergence and international spread of AMR bacteria through the visitors.

Salmonellosis and Campylobacteriosis are leading causes of foodborne diseases (4). With an estimated 100 million individuals falling ill from foodborne infections each year, the Middle East has the third-highest prevalence globally (5). Due to the rapid spread of AMR, fluoroquinolone-resistant *Salmonellae* and *Campylobacter* were declared as high-priority pathogens for research and development of new antibiotics by the World Health Organization (6). Multidrug-resistant (MDR) foodborne pathogens are found widespread across the entire ecosystem and can spread to humans through food, environmental contamination, or direct contact with animals (7). The excessive use of antimicrobials in the food-producing animal industry fuels the emergence and spread of AMR (8). In 2017, approximately 73% of all antimicrobial usage worldwide was reported in animal agriculture (9). Based on recent projections, the global usage of antimicrobials in food-producing animals will further increase by 2030 (10). According to the agricultural census in 2021 by the Palestinian Central Bureau of Statistics (PCBS), there has been a significant growth in the industrial production of broiler chicken meat in Palestine. From 2010 to 2021, a remarkable increase of 128% was reported. This surge in production resulted in a total of 71 million broiler chickens being produced in 2021 (PCBS report in Arabic is available at https://www.pcbs.gov.ps/Downloads/book2606.pdf). The industrialized meat production in Palestine goes along with the excessive use of antibiotics (5, 10) and likely contributes to the spread of AMR.

Early detection and comprehensive understanding of drug-resistant pathogens, including their reservoir, spread, and genetic diversity, are, therefore, crucial to adopt intervention measures to combat AMR (11). This complexity highlights the importance of the One Health approach, which recognizes the interconnection between human, animal, and environmental health and focuses on demonstrating an incremental benefit of closer cooperation between human and animal health and related sectors (12). Implementing this approach in the surveillance, prevention, and control of AMR in food animal production is vital in safeguarding human health and curbing the spread of AMR via the food supply chain. An integrated surveillance response system (iSRS) (13) for AMR should include continuous collection and testing of bacteria from various sources, such as animals, environment, food, and humans, in combination with surveillance tools like whole-genome sequencing (WGS). WGS data enable in-depth investigations into the transmission dynamics of AMR strains that circulate between animals, food, the environment, and humans (14). We used an iSRS approach complemented with WGS to investigate in the Ramallah/Al-Bireh and Jerusalem districts the two leading endemic foodborne pathogens, *Salmonella enterica* and *Campylobacter jejuni* in humans, chickens, and the environment.

## RESULTS AND DISCUSSION

To our knowledge, this is the first integrated One Health survey supported by next-generation sequencing (NGS) that reports on the emergence and spread of *Campylobacter* spp. and *Salmonella* spp. AMR in the Middle East. AMR is a serious global public health concern, and surveillance has historically been concentrated in clinical settings in high-income countries. Outside clinical settings, resistant bacteria can circulate largely undetected in healthy humans, animals, and the environment, particularly in low- and middle-income countries (15). Very few studies exist that study the human-animal-environmental interface simultaneously (16). Our approach combines a One Health study design and WGS to identify the source and spread of foodborne pathogens and their AMR. Integrating WGS data from pathogens collected in the same place and time from humans, animals, and their environment using a One Health iSRS can lead to effective AMR control policies by identifying critical transmission routes and population dynamics (12). NGS technologies, such as Oxford Nanopore Technologies’ MinION sequencing, have revolutionized clinical microbiology (17). Switching to NGS from traditional microbiological methods has a wide range of benefits, such as simultaneous identification of pathogens, assignment of serotypes, and detection of AMR markers. Therefore, integrating WGS-derived information into iSRS will help to design and guide control interventions and support monitoring of their effectiveness leading to reduced morbidity and mortality as well as saving health system resources (18).

### A high positivity rate and distinct risk factors associated with *Salmonella* spp. and *Campylobacter* spp. characterize the broiler chicken production chain in Palestine

**Fig 1:**
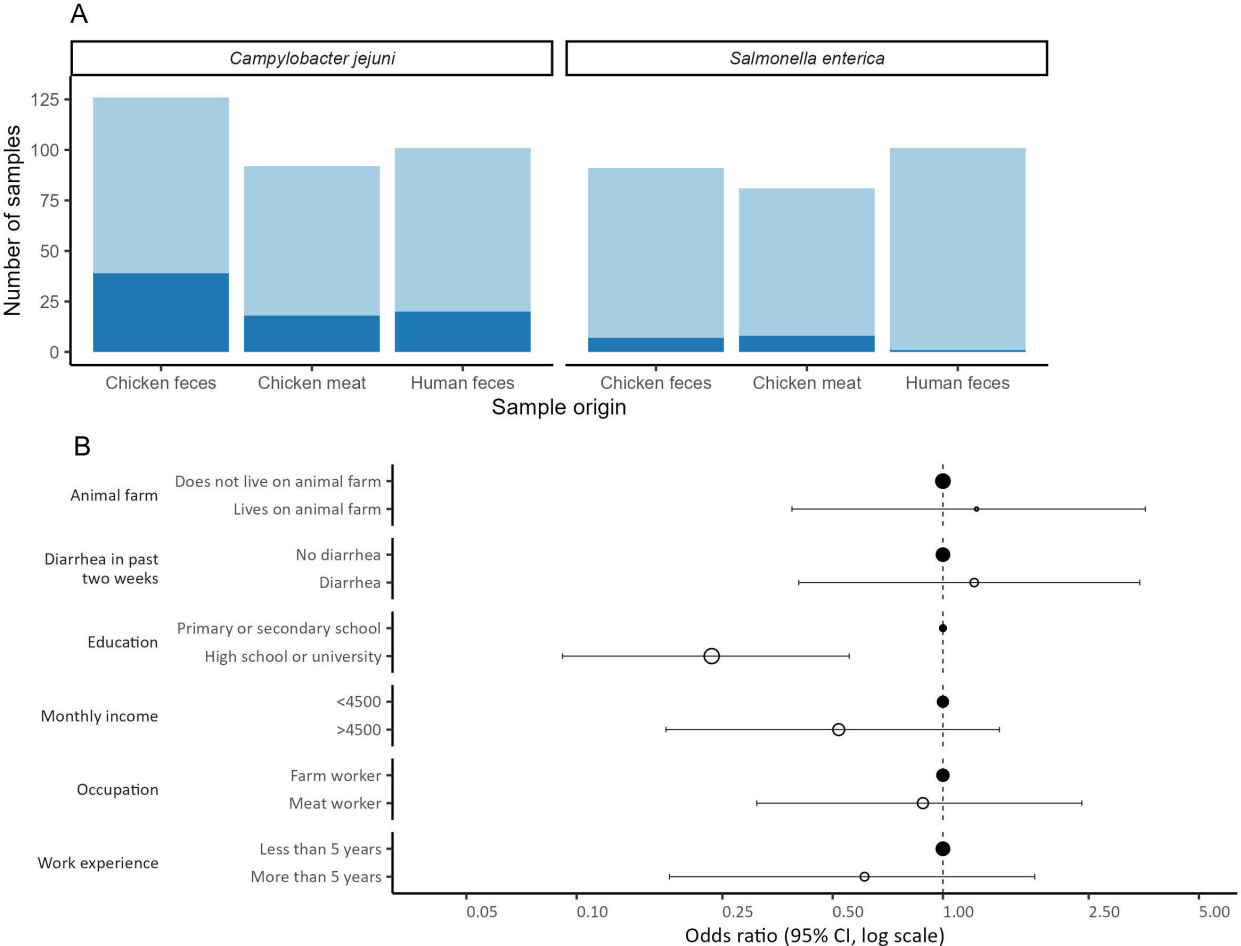
Epidemiology of *C. jejuni* and *S. enterica* in broiler chicken production chain in Palestine. (**A**) Positivity rate of *C. jejuni* and *S. enterica* among samples tested from different sources. (**B**) Univariable analysis of risk factors for *C. jejuni* infection among workers’ sociodemographic characteristics and health status. filled circles are the reference group.

Three hundred and nineteen (319) specimens of *C. jejuni* were collected from chicken manure (*n* = 126), chicken meat (*n* = 92), and human stool (*n* = 101). Two hundred and seventy-three (273) specimens of *S*. *enterica* were collected from chicken manure (*n* = 91), chicken meat (*n* = 81), and human stool (*n* = 101). All samples which were culture positive for *Campylobacter* spp. were identified as *C. jejuni*, and all samples which were culture positive for *Salmonella* spp. were identified as *S. enterica*. The species identification of all positive samples was confirmed by PCR (Fig. 1A). In chicken manure samples, the positivity rate for *C. jejuni* was 30.1% (39/126) and for *S. enterica* 7.7% (7/91). Among chicken meat samples, the positivity rate for *C. jejuni* was 19.6% (18/92) and 9.9% (8/81) for *S. enterica*. Among fecal samples of broiler chicken production workers, we found a positivity rate of 19.8% (20/101) for *C. jejuni* and 1.0% (1/101) for *S. enterica*, respectively. Fig. 1B shows risk factors for infection with *C. jejuni* among workers’ sociodemographic characteristics, with higher education decreasing the risk of infection (OR  =  0.23, *P* < 0.005).

The high positivity rate of *Campylobacter* spp. and *Salmonella* spp. in the investigated samples, especially from chicken manure and meat, highlights the need for better hygiene control in chicken farms and the need for better food safety measures throughout the food production chain. A study on food safety in Palestinian territories found that poor hygiene practices, monitoring system fragmentation, a lack of regulations and controls, and overuse of antimicrobials were obstacles to improving the food safety system (5). These results reflect the socioeconomic challenges in the region, including chronic conflict, population pressure, limited economic development, rapid urbanization, intensive agriculture, and difficulty enforcing policies and regulations (19). In conflict-torn places, collecting samples from the environment can have significant advantages, especially when access to human patients is limited. Environmental samples, such as chicken manure, can provide important information about the presence of foodborne pathogens and AMR, as these pathogens often persist in the environment long after the animals have left (20).

The survey result showed that meat production workers with higher education level have a lower risk of infection with *Campylobacter*. These results are consistent with a recent study that showed that education level was a determinant of zoonotic disease knowledge among workers in the production chain in Palestine and significantly influenced hygiene practices among them (5). Therefore, to address the challenges in food safety, zoonotic diseases, and AMR in Palestine, we recommend several strategies focusing on education, training, and awareness campaigns. Specific groups such as farm workers, slaughterhouse, and meat shop employees, families, and health professionals should be the focus of targeted education and training initiatives to enhance their knowledge and skills.

### A rapid strain and AMR profile turnover are observed in samples positive for *C. jejuni*

**Fig 2:**
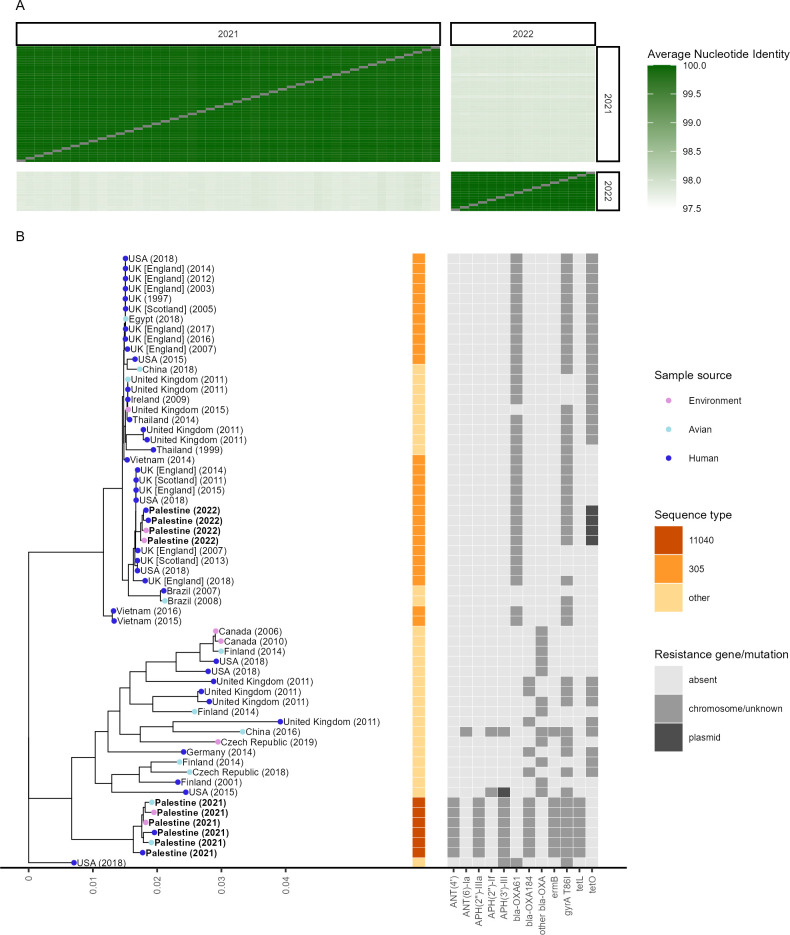
Phylogenetic analysis of *C. jejuni* isolates. (**A**) Average nucleotide identities between all 47 isolates collected in 2021 and all 16 isolates collected in 2022. (**B**) *C. jejuni* phylogenetic tree with 10 representative genomes from Palestine and 51 closely related genomes from different countries collected between 1997 and 2019. MLST-based sequence types are shown for the sequence types represented by Palestinian isolates. The presence of AMR markers is shown in light gray if located in the genome or an unidentified contig or dark gray if located on a plasmid. sample sources are depicted by colored, filled circles. Genomes generated in this study are in bold text. All human isolates collected in 2021 are from broiler chicken production chain workers, while all four human isolates collected in 2022 are from hospitalized gastroenteritis patients.

Regardless of the sample origin, all 47 *C. jejuni* isolates collected in 2021 were highly similar with an average nucleotide identity (ANI) of 99.95% (range: 99.90%–99.98%). The same was observed for 16 *C*. *jejuni* isolates collected in 2022, for which an ANI of 99.98% (range: 99.97%–99.99%) was determined. Comparing the isolates collected in 2021 and 2022 revealed an ANI of only 97.84% (range: 97.80%–97.93%) (Fig. 2A). A phylogenetic tree based on 1,000 single copy genes was created using 10 representative isolates from Palestine (Fig. 2B). For comparison, 29 closely related genomes from Bacterial and Viral Bioinformatics Resource Center (BV-BRC) and 22 genomes with the same sequence type (ST) as identified in PubMLST were added. All 47 isolates collected in 2021 were assigned to ST 11040 by Multi Locus Sequence Typing (MLST). Only one other isolate with the same ST was found in the PubMLST database, a human blood culture isolate collected in 2015 in Israel. These isolates did not match closely with any known genomes and showed a distinct AMR pattern. All samples collected in 2021 consisted of a single chromosome with a median length of 1,670,867 base pairs (range: 1,670,766–1,671,976 bp) and had identical drug resistance gene patterns, consisting of the aminoglycoside resistance genes *APH(2′′)-IIIa*, *ANT(4′*), and *ANT (6)-Ia*, OXA β-lactamase *bla-OXA184*, macrolide resistance gene *ermB*, quinolone resistance mutation T86I in *gyrA*, and tetracycline resistance gene *tetL*. No plasmid was detected. The closest WGS we found had an ANI of only 98.39% (range: 98.27%–98.44%). The co-resistance conferred by the combination of AMR markers to both aminoglycosides and macrolides is worrisome, as these drugs are typically the first line of treatment for human cases (21). The 16 isolates collected in 2022 were assigned to ST 305, which has a total of 95 entries in PubMLST. The majority of sequences with available WGS data (53.1%, 17/32) were submitted by the United Kingdom, while only one isolate originated from the Middle East. These isolates consisted of a chromosome with a median length of 1,677,345 bp (range: 1,677,331–1,677,357 bp) and a plasmid with a median length of 51,587 bp (range: 51,584–51,589 bp). Isolates collected in 2022 had a resistance gene pattern consisting of OXA β-lactamase *bla-OXA61* and quinolone resistance mutation T86I in *gyrA* on the chromosome and the plasmid-encoded tetracycline resistance gene *tetO*. The *C. jejuni* isolates collected in 2022 did not have AMR markers conferring resistance to aminoglycosides or macrolides. Establishing an iSRS to address large annual fluctuations in bacterial strains and resistance characteristics requires enhanced collaboration and partnerships among relevant stakeholders (22). Implementation of standardized protocols for data collection, testing, and reporting is essential, supported by a robust data management system for centralized data collection and analysis (22). Regular monitoring activities also play a vital role in observing dynamic patterns, while collaborative research efforts can help identify factors that contribute to these changes (23). In addition, capacity building through introducing new laboratory techniques such as WGS, training programs, and effective sharing of information and communication among stakeholders is pivotal (18, 24). If successfully implemented, these approaches could result in an immediate response and intervention to effectively address emerging AMR threats. A recent study identified Palestine as a global hotspot for veterinary antimicrobial consumption (10), underscoring the problem of antibiotic overuse in intensive animal production. This issue is particularly prevalent in broiler production, where lower levels of biosecurity measures lead to heavy reliance on antibiotics. This excessive use could be a contributing factor to the rapid shift in the *C. jejuni* strain observed between 2021 and 2022.

### The MDR isolates *S. enterica* serovar Muenchen are found across the entire broiler chicken production chain

**Fig 3:**
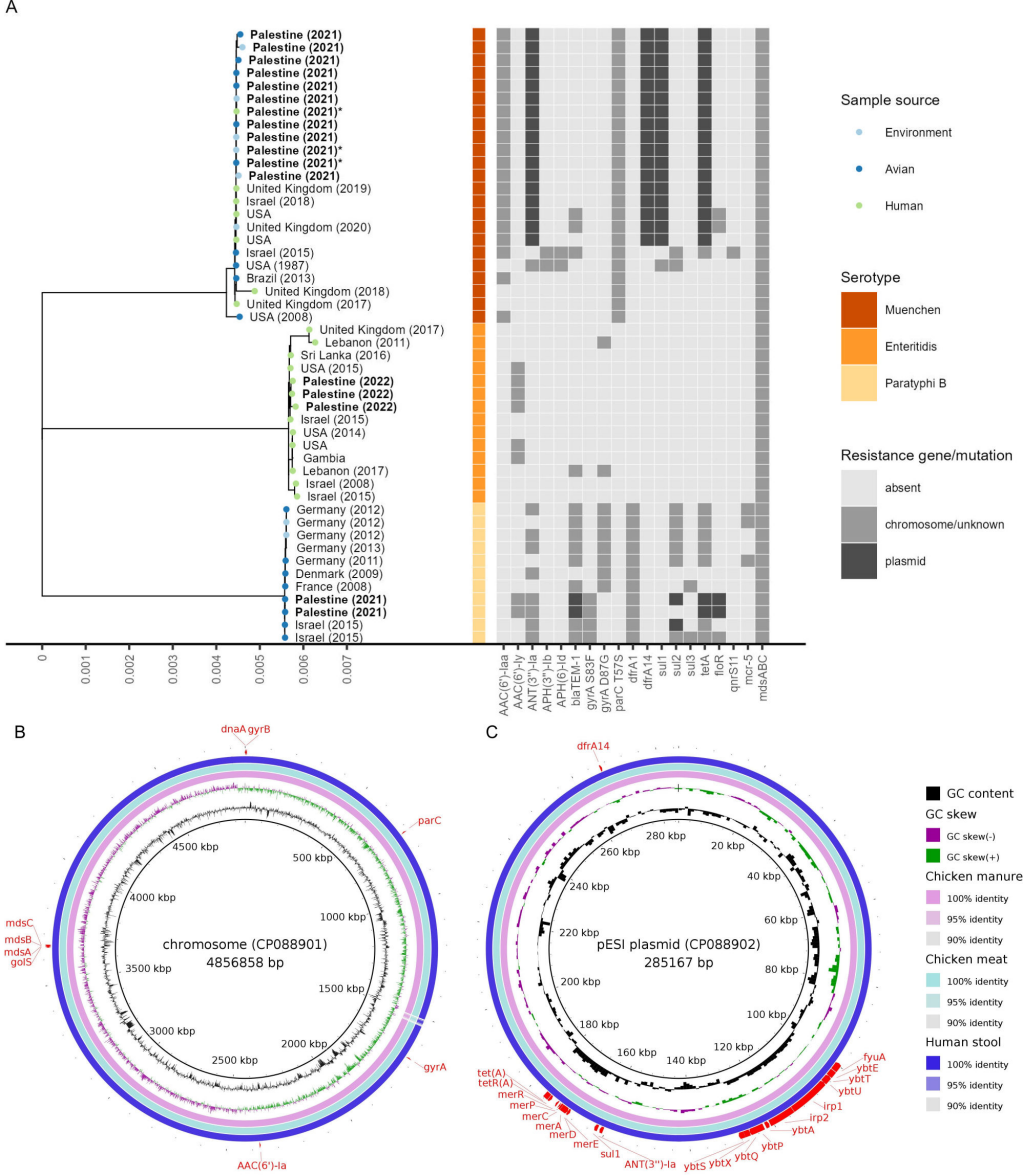
Phylogenetic analysis of *S. enterica* isolates. (**A**) Phylogenetic tree for *S. enterica* with 17 genomes from Palestine and 31 closely related genomes belonging to the three serotypes found in this study from different countries collected between 1987 and 2020. The presence of AMR markers is shown in light gray if located in the genome or an unidentified plasmid or dark gray if located on a plasmid. Sample sources are depicted by colored, filled circles. Genomes generated in this study are in bold text. Genomes used for Fig. 3B and C are marked with an asterisk. Comparison of Palestinian *S. enterica* serotype Muenchen chromosome (**B**) and pESI plasmid (**C**) from chicken manure and human feces as well as meat with a clinical isolate from Israel collected in 2018. The presence and location of antibiotic-resistance genes are shown in red. The human isolate collected in 2021 is from a broiler chicken production chain worker, while all three human isolates collected in 2022 are from hospitalized gastroenteritis patients.

A phylogenetic analysis based on 1,000 single-copy genes for *S. enterica* revealed that 12 out of 14 *S*. *enterica* isolates collected in 2021 were assigned to *S. enterica* serovar Muenchen (Fig. 3A). These 12 isolates had a median chromosome length of 4,857,056 bp (range: 4,856,898–4.857,070 bp) and a median plasmid length of 285,076 bp (range: 285,070–285,085 bp). The isolates show a high similarity across all samples with an ANI of 100.00% (range: 99.99%–100.00%). The two other isolates were sampled from chicken meat in 2021 and assigned to *S. enterica* serovar Paratyphi B. The two *S*. *enterica* serovar Paratyphi B isolates had chromosome lengths of 4,697,034 bp and 4,697,043 bp and chromosome encoded aminoglycoside resistance genes *AAC(6′)-Iy* and *ANT(3′′)-Ia*, quinolone resistance mutation S83F in *gyrA*, trimethoprim resistance gene *dfrA1*, and the *mdsABC* efflux pump. Additionally, they have the plasmid-encoded β-lactamase *blaTEM-1*, phenicol resistance gene *floR*, sulfonamide resistance gene *sul2*, and tetracycline resistance gene *tetA*. They were closely related to *S. enterica* serovar Paratyphi B isolates collected from environmental and avian sources in Europe and Israel. Additionally, three confirmed *S. enterica* clinical isolates obtained from humans in 2022 were added as positive controls. These three isolates had a median chromosome size of 4,720,112 bp (range: 4,720,107–4,720,159 bp), a plasmid with a median length of 37,698 bp (range: 37,698–37,699 bp) as well as a plasmid with a median length of 59,372 bp (range: 59,372–59,373 bp). The *S. enterica* serovar Enteritidis isolates contain the chromosome-encoded aminoglycoside resistance gene *AAC(6′)-Iy* and the *mdsABC* efflux pump. They were closely related to *S. enterica* serovar Enteritidis strains isolated from humans globally. All 12 isolates assigned to *S. enterica* serovar Muenchen shared an identical pattern of molecular markers for AMR with resistance markers, including the aminoglycoside resistance gene *AAC(6′)-Iaa*, the fluoroquinolone resistance mutation T57S of *parC*, and the *mdsABC* efflux pump encoded on their chromosome (Fig. 3B). Additionally, on the 285,077 bp (range: 285,070–285,085 bp) megaplasmid that was 99.99% (99.97–100.00) similar to “plasmid of emerging S. Infantis (pESI)” described from Israel (25), aminoglycoside resistance gene *ANT(3′′)-Ia*, sulfonamide resistance gene *sul1*, tetracycline resistance gene *tetA*, and trimethoprim resistance gene *dfrA14* are located (Fig. 3C).

Our study shows that the MDR *S. enterica* serovar Muenchen isolates are found in the entire chicken meat production chain. Among the *S. enterica* serovar Muenchen isolates, we found genotypical resistance markers to at least five classes of antibiotics, including aminoglycosides, fluoroquinolones, aminocoumarines, sulfonamides, and tetracyclines. Given the significant clinical importance of *S. enterica* infections, with a global annual incidence of over 27 million cases of enteric fever (26) and 78.7 million cases of gastroenteritis (4), the AMR monitoring as part of a One Health iSRS is of utmost importance.

All 12 isolates identified as *S. enterica* serovar Muenchen in our study showed a close relationship to an emerging clinical isolate reported in Israel (25). In addition, this study identified 19 additional *S. enterica* serovar Muenchen isolates from the United States, the United Kingdom, and South Africa containing the pESI plasmid with high genetic similarity (25). These *S. enterica* serovar Muenchen isolates were obtained from human clinical samples or avian sources. This noteworthy similarity among globally sourced isolates is likely to be attributed to the widespread and synchronized dissemination of breeding stocks contaminated with the bacteria due to the centralized sourcing practices and international trade. Similar patterns have been observed for *S. enterica* serotype Enteritidis (27), highlighting the role of global trade in facilitating the spread of such MDR bacterial strains.

The researchers from Israel noted an increasing prevalence of an MDR strain of *S. enterica* serovar Muenchen among their clinical *Salmonella* spp. isolates (25). These isolates displayed significant sequence similarity to the isolates described in our study. This suggests that there may be a regional spread of this particular MDR strain. This could be due to importing some of the raw materials used in animal feed production from Israel, intersecting distribution networks, and sharing the same ecosystem (19, 28). Moreover, the prolonged and intense conflicts in the Palestinian territories contribute to a weak control system, which leads to cross-border smuggling (5, 28). The discovery of a dominant MDR strain emphasizes the need for continuous monitoring and open data sharing in real time to understand the epidemiology of AMR across borders and political systems.

## MATERIALS AND METHODS

### Study design, setting, and participants

This cross-sectional study was performed in Ramallah/Al-Bireh and Jerusalem governorates of the central West Bank, Palestine. Between June and October 2021, 557 samples were collected, and 284 samples were tested for the presence of *Campylobacter* and 273 samples for *Salmonell*a. The samples came from abattoirs (*n* = 50, fresh chicken meat), large-scale broiler chicken farms with single batch at a time (all-in and all-out) system (*n* = 91), chicken manure, and asymptomatic chicken meat production workers (*n* = 101, fecal samples). In August 2022, an additional 35 chicken manure samples were collected from the same farms addressed in 2021 to investigate the persistence or replacement of *C. jejuni* strains over time. Furthermore, four *C. jejuni* isolates and three *S*. *enterica* isolates were isolated from gastroenteritis patients in 2022 after confirmation by Vitek 2 and PCR, preserved in the hospital laboratory to be used as positive control isolates at one of the targeted region’s hospitals to compare the pathogens isolated in our study to those reported in the hospitals. To investigate the risk factors for the presence of *Salmonella* spp. and *Campylobacter* spp. along the broiler chicken production chain, a questionnaire in Arabic was used to collect information on workers’ sociodemographic characteristics and health status related to infection with gastroenteritis infections. All sampling sites were selected using a random sampling approach and using authorities’ records about farms, abattoirs, meat stores, and workers. All study participants signed a written consent form.

### Microbiological laboratory procedures

The fecal samples from humans and chicken manure were grown directly on Campylobacter Selective Agar (Oxoid, UK) for *Campylobacter* selection and Xylose-Lysin-Desoxycholat Agar (Oxoid, UK), MacConkey (Oxoid, UK), and Sheep Blood Agar Base (Oxoid, UK) for *Salmonella* selection. Agar plates were incubated at 37°C and checked for the presence of colonies after 24–48 hours. The chicken meat samples were pre-processed according to the ISO 10272-1:2017 guidelines for *Campylobacter* and ISO 6579-1:2017 guidelines for *Salmonella*. From all samples, suspected colonies for each of the species were selected for microbiological confirmation by the Vitek 2 NH ID card for *Campylobacter* or Vitek 2 GN ID card for *Salmonella* using the Vitek 2 compact automated system (BioMerieux, France). All isolates which were confirmed as *Salmonella* or *Campylobacter* by the Vitek 2 microbial testing system were subjected to further confirmation by PCR. Molecular species identification for *Campylobacter* spp. was performed using the PCR protocol published by Nayak et al. (29), while for *Salmonella* spp., the procedure published by Paião et al. was used (30).

### DNA extraction and WGS

From a total of 65 *C*. *jejuni* and 19 *Salmonella* spp. isolates, DNA was extracted using QIAamp DNA Mini Kit (Qiagen, Germany) and Quick-DNA Fungal/Bacterial Miniprep Kit (Zymo Research, USA), respectively. Extracted DNA was shipped to the Swiss Tropical and Public Health Institute. DNA was quantified with the Qubit dsDNA HS Assay Kit (Invitrogen, Germany). Samples with DNA concentrations >33 ng/µL were selected for WGS using MinION platform (Oxford Nanopore Technologies, UK). The sequencing library was prepared according to the manufacturer’s instructions using the Native Barcoding Kit 96 and loaded onto the R10.4 flow cell and sequenced on the MinION Mk1C using super-accurate basecalling.

### Bioinformatics and statistical analysis

*De novo* assembly was conducted using Flye 2.9.1 (31) and Trycycler v0.5.3 (32) at the scientific computing core facility of the University of Basel. The BV-BRC was used for annotation and phylogenetic analysis of the assemblies. The assemblies were annotated using the RAST 2.0 toolkit (33). Only assemblies with less than 10 contigs were considered for further analysis. ST and serotype of assembled contigs were determined using MLST (34) and SeqSero (35), respectively. Phylogenetic trees were generated by aligning protein and nucleotide sequences using MUSCLE (36), MAFFT (37), and RAxML (38). The 20 most closely related genomes from BV-BRC were selected for each *C. jejuni* ST. Additionally, all available WGS for the respective ST were obtained from the public databases for molecular typing and microbial genome diversity (PubMLST). The 10 most closely related genomes from BV-BRC were selected for each *S. enterica* serotype. Additionally, four selected *S. enterica* serotype Muenchen genomes containing the pESI plasmid were obtained from the National Center for Biotechnology Information (GCA_008248485, GCA_009444355, GCA_010825065, GCA_019543295). *C. jejuni* and *S.enterica* genomes were filtered to contain high-quality genomes with available data for collection year, country, and sample origin.

Closely related isolates from the Middle East collected after 2008 were selected for *S. enterica*. Genome-wide ANIs were determined with FastANI (39). Resistome analysis was performed using CARD (6.0.0) (40) and ResFinder (4.2.2) (41). The comparison of *S. enterica* serotype Muenchen genomes was visualized using BRIG (42). Risk factors for *C. jejuni* infections among study participants were calculated using median-unbiased estimates to calculate the univariate odds ratio using the R software environment version 4.2.2 and the package epitools.

## Data Availability

The data that support the findings of this study are available from the authors upon reasonable request. The genome assemblies and raw sequence reads are available on Genbank under BioProjects PRJNA942086 (*S. enterica*) and PRJNA942088 (*C. jejuni*).
